# Formulation concepts in the care of children and adolescents identifying as transgender or gender diverse

**DOI:** 10.1177/10398562251362739

**Published:** 2025-08-02

**Authors:** Jillian Spencer, Roberto D’Angelo, Patrick Clarke

**Affiliations:** Greater Brisbane Clinical School, 1974 University of Queensland, Brisbane, QLD, Australia; 92899 Institute of Contemporary Psychoanalysis, Los Angeles, CA, USA; Faculty of Health and Medical Science, 1066 University of Adelaide, North Adelaide, SA, USA

**Keywords:** gender distress, gender-affirming care, formulation, children, adolescents

## Abstract

**Objective:**

To assist mental health clinicians to develop a biopsychosocial formulation for children and adolescents with gender distress.

**Conclusions:**

Various biological, psychological, and social factors, developmental disorders and adverse experiences, may contribute to a child claiming a trans identity. Factors relevant to the individual child or adolescent should be encapsulated in a formulation to guide therapeutic approaches.

Our field is currently grappling with seemingly irreconcilable conceptualisations of gender dysphoria in children and young people. According to the most-cited guidelines developed by WPATH^1^ and the Royal Children’s Hospital in Melbourne,^2^ being trans or gender diverse is part of the natural spectrum of human diversity. According to these groups, affirmation of a young person’s gender via social, medical or surgical interventions is helpful and even ‘medically necessary’.^1^

It is well established that there are high rates of mental health problems in children and adolescents claiming a trans identity.^3^ According to the gender-affirming model, these problems are largely a consequence of stigma and social exclusion.^1^

According to this perspective, one would expect a child’s gender distress, along with any comorbid mental health problems, to improve or resolve as a consequence of gender-affirming interventions. However, systematic reviews conclude the evidence for such benefits is weak and subject to bias and confounding.^4,5^ In the face of this emerging evidence, gender-affirming clinicians continue to recommend this approach but increasingly for different reasons. Two recent papers have questioned the expectation of gender interventions resulting in improvements to mental health and functioning, and have suggested that treatment objectives include autonomy, ‘appearance congruence’ and ‘embodiment goals’.^6,7^

However, psychologically informed clinicians view gender-dysphoria as a multi-determined phenomenon occurring in the context of complex psychological, developmental, biological, relational and social factors (See Box 1 for a case example). Following a 4-year review of the relevant literature and extensive community consultation, NHS England’s commissioned Cass Review^4^ concluded: ‘there are many pathways into gender dysphoria and many pathways out’. The final report recommended prioritising psychosocial interventions within mainstream mental health services and found no convincing evidence that the affirming model improved the mental health of minors.

**Box 1: Case example**

A 12-year-old girl presented to a Child and Adolescent Mental Health Service suffering symptoms of Major Depression and reporting a trans identity. She had experienced sustained bullying throughout primary school and was noted by the clinician to demonstrate mild social oddity. Her parents worked long hours, and she was spending extended periods of time online where she had developed connections with other young people reporting a trans identity. Extended assessment confirmed the presence of a mild autism-spectrum disorder and identified a history of sexual abuse from an uncle. The clinician’s initial formulation considered that feelings of disconnection from peers and family may have led to ongoing low self-worth and a sense of hopelessness about establishing enduring positive connections. A history of sexual abuse may have led to anxiety about her developing body and the possibility of sexual attention and contact. Features of an autism-spectrum disorder were considered to underpin a tendency towards rigid thinking regarding male and female roles and a fixation on transition as a solution to her complex problems. A trans identification was considered to offer an avenue for gaining social connection whilst allowing the avoidance of anxiety related to the risks and expectations of becoming a woman.

If Australia shifts to a treatment approach in line with the research evidence and consistent with the recommendations of the Cass Review, clinicians within mainstream child mental health services will need to deliver psychosocial approaches of care guided by a formulation. The authors have extensive clinical experience with young people reporting a trans identification. Drawing upon our clinical experience, and the research literature, in this article we consider seven factors which might contribute to the development of trans identification: body dissatisfaction; social reinforcement; gender non-conformity and homophobia; political trans identification; adverse childhood experiences; autism-spectrum disorders; and family dynamics. We hope this will provide clinicians with a basic framework for thinking holistically about young people with gender distress and will enable them to provide more targeted and patient-specific psychosocial care.

## Body dissatisfaction

Rates of body dissatisfaction are high amongst normal adolescents.^8^ Girls entering puberty early are at higher risk of experiencing anxiety about their bodies^9^ due to feeling different from peers and being developmentally unready to manage male attention, particularly for those who develop larger breasts. Boys are also vulnerable to poor body image with the muscularity of male body representations in the media presenting a largely unattainable body ideal that can foster feelings of inadequacy.^10^ Young people claiming a trans identity frequently express hatred, disgust or shame about aspects of their body, particularly the sexed aspects such as breasts, body hair, voice pitch, etc. In the authors’ experience, trans identification can provide an explanation which makes sense of this distress and provides a (fantasied) solution. Some adolescents may come to believe that gender interventions will make them more attractive.

For adolescents struggling with psychosexual development, managing sexual feelings and sexual attention can be challenging. Reporting gender distress as part of a trans identity may also be a socially acceptable way to avoid sexual contact whilst maintaining belonging to a group.

## Social reinforcement and the online world

The social environment in which children and adolescents live provides messaging that views trans identification as a positive development.^11^ Adolescents are particularly prone to social influence due to the strong developmental need to belong. Claiming a trans identity can provide entry to a welcoming online social network.

A key part of the transgender political movement has been to celebrate a transgender identity with brightly coloured symbols and LGBTIQA+ ‘Pride’ celebrations, including the entire month of June.^12^ Consequently, children claiming a transgender identity may receive a high level of attention, perceived special status and protection against criticism or social rejection. Special measures taken by others to demonstrate support for the child’s claimed identity may make it difficult for the child to desist, fearing people may resent having made such efforts.

## Gender non-conformity and homophobia

Children naturally vary in their level of comfort with traditional gender stereotypes. Sex non-conformity and cross-sex identification in childhood is associated with an increase in the likelihood of later same-sex attraction.^13^ Gender non-conformity is normal, however, parents may misinterpret a child’s gender non-conformity as indicative of an enduring trans identification. Observational studies indicate that the majority of children demonstrating cross-sex identification will become comfortable with their sexed body if allowed to undergo puberty and develop normally into adulthood.^14^ Despite the recent social changes that have led to acceptance of same-sex attracted people in our culture, homophobia remains prominent in schools.^15^ Adolescents struggling with discomfort or shame about same-sex attraction may pursue gender transition to avoid homosexuality. Indeed, this is encouraged as a solution to same-sex attraction in certain Middle Eastern countries.^16^ Staff working at the UK’s Tavistock Gender Identity Service raised concerns that discomfort with gender non-conformity, fundamentally a form of homophobia, may cause a parent to pursue gender transition to avoid their child becoming homosexual.^17^

## Political trans identification

A trans identification may be considered part of a social justice movement which seeks to undermine traditional power structures of ‘cisheteronormativity’.^18^ Queer theory critiques the normative status of heterosexuality and suggests the impossibility of any ‘natural’ sexuality.^19^ It mainly examines the tyranny of sexual and gender normativity, however, homonormativity, whiteness, family values, marriage, monogamy and Christmas have also been subject to sustained critique.^20^ Adolescents may adopt a trans identity to contribute to this political movement, and to avoid stigma associated with being part of a dominant, oppressive class. Adolescents and young adults are often drawn to countercultural groups due to their developmental process of individuation, exploration and the challenging of parental norms.

## Adverse childhood experiences

Trans identification can be a way for adolescents to make sense of traumatic sequelae. People who have experienced developmental trauma often experience feelings of defectiveness or badness; a sense that something is seriously wrong with the self. There are often feelings of shame associated with perceived defects in the self and these can be projected onto the body. The body becomes the source of distress and the solution therefore involves eliminating aspects of the body that feel ‘wrong’. A trans identity can involve the fantasy that the badness can be eliminated and a new identity will be free of internal pain. Similarly, a child who is socially isolated and unable to form friendships, perhaps because of a history of adverse experiences, such as deprivation or abandonment, may seek an explanation for feeling disconnected from others. The fantasy may be that if they transition, they will be more loveable and able to make friends, providing longed-for acceptance. Young people who have experienced developmental trauma are often excessively compliant, leaving them particularly vulnerable to social influence.

Other forms of abuse can also underpin a trans identification in young people. Boys witnessing or subject to male violence may fear their own masculinity and aggression, driving them to seek transition to eliminate perceived dangerous maleness. Sexual abuse can shape the experience of the body, of sexual desire and of gender. Sexual arousal during sexual abuse is common and frequently leads to significant internal conflict and shame.^21^ This may present as repulsion for the genitals or as a dread of feeling sexual desire. Girls who have experienced or witness sexual abuse may fear further sexual violation as they become aware of the pervasive objectification of women in our culture. Transitioning may provide a solution to evade male attention.

## Autism-spectrum disorders

Children with autism-spectrum disorders often struggle with change and are therefore often struggle with the physical and social changes of adolescence. They may seek to prolong childhood due to discomfort with their emerging secondary sex characteristics. Autistic traits may also lead to rigidity in thinking, and reduced abstract thinking, which may make a child or adolescent susceptible to viewing gender stereotypes quite literally, for example, ‘I don’t like traditional masculine activities, so I must not be a boy'. Unsurprisingly, features of autism are common in children claiming a trans identity.^22^ Autism-spectrum disorders affect social development and communication, which can result in a child feeling different from their peers and experiencing rejection or bullying. This can lead to the child embracing a trans identity as an explanation for feeling different. The trans identity may facilitate much wanted social connection. A social justice framework amongst trans-identified children with autistic traits can provide a comforting structure for how to connect with peers as it provides ‘off the shelf’ acceptable viewpoints and a set of common-interest topics for conversation.

For unclear reasons, possibly related to a preoccupation with parts of objects rather than an appreciation for the whole, in addition to a reduced understanding of relationships, people with autism-spectrum disorders, especially males, are more prone to developing paraphilias, such as arousal from cross-dressing (transsexualism) or arousal from imagining oneself as a woman (autogynephilia).^23^ A consequence of medical affirmation is reduced or loss of libido and sexual functioning.^24^ Although initially this may be regarded as a relief from unwanted urges, ultimately it is likely to add to long term regret over loss of sexual pleasure and the ability to function normally in an intimate relationship.

## Family dynamics

Parents’ individual psychologies and developmental histories may be a contributing factor to how their children experience gender. For example, a mother with a history of physical abuse may be anxious about a male child’s potential for violence and may inadvertently communicate that masculinity is undesirable. Similarly, a history of sexual abuse can leave parents anxious about a female child’s risk of abuse and this may be subtly communicated to the child, generating discomfort about her biological sex and the prospect of becoming a woman. More directly, parental preferences for a child of a particular sex may influence a child’s experience of their sexed body and reinforce cross-sex behaviours through parental attention and praise. Vulnerable parents may obtain social secondary gain by unconsciously or consciously cultivating or positively reinforcing a trans identity in their child in the context of media culture promoting trans-identified children^34^ and gender-affirming parent support groups.

Parental disengagement or neglect can occur when the couple is experiencing relationship dysfunction, or when the parent(s) are preoccupied with other traumas and losses. This may lead a child to seek guidance, comfort or engagement from online entities. At the same time, claiming a trans identity is a powerful strategy to recapture parental focus. On the other hand, parental emotional enmeshment may contribute to a child claiming a trans identity to individuate and express their anger at being burdened by a parent’s emotional needs. In both cases, claiming a trans identity can serve the dual function of demarcating the child’s separateness whilst also keeping them at the centre of the family’s attention.

Separated parents may struggle to form a united parental alliance, and this may render one parent vulnerable to affirming a child’s claimed gender identity to maintain a connection with the child.

The false narrative of higher suicide risk amongst children and adolescents who are not affirmed,^4,25^ and the fear generated by online influencers who encourage children to reject parents who don’t affirm, can create a reversal in power dynamics such that parents become fearful of setting appropriate limits on the child across a range of domains. This can, in turn, lead to a child paradoxically dominating the parent/child relationship. Sibling relationships can also be significant. Trans identification may be an attempt to emulate or differentiate from a sibling who is perceived to be more successful or favoured by their parents.

## Conclusions

Should Australia follow the recommendations of the UK Cass Review and shift the care of children claiming a trans identity to mainstream child and youth mental health services, mental health clinicians within those services will benefit from considering a wide range of aetiological and maintaining factors contributing to their patient’s presentation. A detailed, careful and evolving formulation is recommended to guide therapeutic interventions.
